# Circ_0008305‐mediated miR‐660/BAG5 axis contributes to hepatocellular carcinoma tumorigenesis

**DOI:** 10.1002/cam4.3657

**Published:** 2021-01-22

**Authors:** Fuguo Yan, Bin Fan, Jianchu Wang, Wang Wei, Qianli Tang, Libai Lu, Zongjiang Luo, Jian Pu, Shan‐Shan Yang

**Affiliations:** ^1^ Department of General Surgery The Xinchang Hospital Affiliated to Wenzhou Medical University Xinchang China; ^2^ Department of Hepatobiliary Surgery The Central Hospital of Enshi Autonomous Prefecture Enshi China; ^3^ Department of Hepatobiliary Surgery Affiliated Hospital of Youjiang Medical University for Nationalities Guangxi Zhuang Autonomous Region China; ^4^ Department of Pediatrics Huai’an Second People’s Hospital The Affiliated Huai’an Hospital of Xuzhou Medical University Huai’an China

**Keywords:** BAG5, circ_0008305, hepatocellular carcinoma, miR‐660

## Abstract

Increasing circRNAs have attracted a lot of attention because of their significant biological effects in many diseases. It has been reported that circ_0008305 can modulate lung cancer progression. However, the association between circ_0008305 and hepatocellular carcinoma (HCC) needs to be well explored. In this current research, we studied the molecular function and potential mechanism of circ_0008305 in HCC progression. First, it was demonstrated that circ_0008305 was greatly increased in HCC tissues and cells. Moreover, we observed silencing circ_0008305 markedly repressed HCC cells in vitro growth and reduced tumor growth in vivo. Additionally, it was identified that circ_0008305 can act as a sponge of miR‐660 while miR‐660 targeted Bcl‐2‐associated athanogene 5 (BAG5). BAG5 belongs to a member of BAG family and it is involved in multiple diseases. We reported that circ_0008305 contributed to the inhibition of miR‐660, which resulted in an upregulated expression of BAG5 in HCC. Subsequently, rescue assays were conducted and it was indicated that loss of BAG5 reversed the effects of miR‐660 inhibitors on HCC partially. To sum up, it was illustrated by our study that circ_0008305‐mediated miR‐660‐5p/BAG5 axis triggered HCC progression, which could provide a novel insight on the underlying mechanism of HCC progression.

## Graphical abstract

circ_0008305 was greatly increased in HCC tissues and cells. circ_0008305 can act as a sponge of miR‐660. miR‐660 targeted Bcl‐2‐associated athanogene 5 (BAG5).

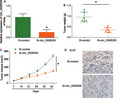

## 1 INTRODUCTION

Hepatocellular carcinoma (HCC) is becoming a prevalent cancer worldwide.^1^ A great number of cancer‐related death has been caused by this malignant tumor every year.^2^ As well established, common therapy for HCC are chemotherapy and surgery. During past years, great advances are made in HCC. Nevertheless, the prognosis of HCC is still poor with a lower 5‐year survival rate because of tumor recurrence and metastasis.^3^ Therefore, it is significant to identify the novel biomarkers for HCC.

In recent years, circRNAs are attracting much attention. CircRNAs are noncoding RNAs with little potential of protein coding.^4^ CircRNAs, covalently closed transcripts, can be derived from back splicing of precursor mRNA.^5^ CircRNAs can be involved in gene expression via sponging microRNAs or showing specific interactions with other molecules to regulate their function.^6^, ^7^, ^8^ Meanwhile, increased studies indicated that circRNAs can be closely correlated with various disease such as neurological disorders, vascular diseases, and carcinomas.^9^, ^10^, ^11^ For example, circ_100395 can regulate miR‐1228 and TCF21 to repress the progression of lung cancer.^12^ Circ_100876 induce breast cancer cell proliferation and metastasis by sponging miR‐361‐3p.^13^ In bladder cancer, circ‐RNA BCRC‐3 can repress proliferation via regulating miR‐182‐5p and p27.^14^ Taken these together, circRNA–miRNA–mRNA interaction could act as a crucial regulatory network in cancer development.

In current work, we focused on the effects of circ_0008305 on HCC cell proliferation, migration, and invasion. Circ_0008305 was obviously elevated in HCC and effectively contributed to HCC cell progression. We demonstrated that loss of circ_0008305 suppressed the HCC progression through regulating miR‐660/BAG5 axis. Hence, it was suggested the circ_0008305/miR‐660/BAG5 axis might be a novel target for HCC.

## 2 MATERIALS AND METHODS

### 2.1 Tissues specimens

Thirty paired HCC specimens and matched noncancerous tissues specimens diagnosed between 2015 and 2018 were obtained from tumor surgical resection in our hospital, and we got the approval of the Medical Ethics Committee. Thirty normal liver tissues collected (hepatic hemangioma patients) were acquired between 2015 and 2018. Informed consent was provided by the enrolled patients. Tissue samples were maintained at −80°C for future assays.

### 2.2 Cell culture and transfection

HCCLM3, MHCC97L, Hep3B, HepG2, and THLE‐3 cells were obtained from ATCC and maintained in DMEM medium (Hyclone, Logan) added with 10% FBS. Cells were incubated at 37°C with 5% CO_2_. miR‐660 mimics, inhibitors, BAG5, and circ_0008305 siRNA were obtained from Genepharma. Transfection was carried out using the Lipofectamine™ 3000 (Invitrogen).

### 2.3 Assessment of cell viability

Cell proliferation assay was performed using CCK‐8 kit (Dojindo). Cells (3 × 10^3^ each well) were seeded into 96‐well plate. CCK8 were added to the cells for 2 h. We measured the spectrometric absorbance using a microplate reader at 450 nm at various hours.

### 2.4 Apoptosis analysis

Annexin V‐FITC/PI double staining was employed to analyze apoptotic cells using Annexin V‐FITC Apoptosis Detection Kit (BioVision). We collected cells and washed the cells using cold‐PBS. Afterwards, cells were stained using Annexin V‐FITC and PI for 10 min. Then, the stained cells were analyzed using a BD FACSCanto II.

### 2.5 Cell cycle evaluation

Cells were fixed using 70% ethanol and then, stored at 4°C for a whole night. Cells were rehydrated by PBS for 10 min and cells were stained by PI contained with 50 μg/mL PI, 2 μg/mL DNase‐free RNase A, and 0.2% NP‐40 in PBS for 15 min. In the end, cell cycle was analyzed by BD FACSCanto II (BD Biosciences).

### 2.6 Transwell assay

Cell migration and invasion assay were tested by transwell chambers. To do migration assay, cells were seeded into the upper chamber with serum‐free medium. After 24 h, the cells on the lower chamber with 10% FBS were fixed using 4% paraformaldehyde and stained using 0.1% crystal violet. To perform the invasion experiment, only the upper chamber was pre‐coated by Matrigel (BD Biosciences).

### 2.7 Western blotting assay

Cell lysate was prepared by RIPA buffer. Protein sample was separated on 10% SDS–PAGE and nitrocellulose membranes were used to transfer the protein. Then, antibodies against BAG5 and GAPDH were purchased from Cell Signaling Technology. HRP‐conjugated goat anti‐mouse IgG or anti‐rabbit IgG was purchased from Cell Signaling Technology. We visualized the labeled proteins using Immobilon Western Chemiluminescent HRP Substrate kit (Millipore).

### 2.8 qRT‐PCR analysis

RNA was isolated using RNeasy mini kit (Qiagen). Single‐stranded cDNA was generated by PrimeScript^™^ RT Master Mix (Takara). qRT‐PCR was performed in LightCycler 96 (Roche) using a LightCycler FastStart DNA Master SYBR Green I (Roche). Validated qRT‐PCR primers were listed in Table 1. Relative data were obtained by 2^−ΔΔCt^.

**TABLE 1:** Primers for real‐time PCR

Genes	Forward (5’−3’)	Reverse (5’−3’)
GAPDH	ATGTCGTGGAGTCTACTGGC	TGACCTTGCCCACAGCCTTG
Circ_0008305	CGGGCTTTGCCATCAATACC	TTGGCCTTGACAGAATCCAG
miR−660 U6 BAG5	TACCCATTGCATATCGG CTCGCTTCGGCAGCACA TGTCCCCGGGTTTAGGGGTGTTC	GTGCAGGGTCCGAGGT ACGCTTCACGAATTTGCGT TTCACAAGCACTGTCCCGCCC

### 2.9 Dual‐luciferase reporter assay

The possible binding sites in circ_0008305 and BAG5 3′‐UTR were predicted using circinteractome (https://circinteractome.nia.nih.gov/) and TargetScan7.1 (http://www.targetscan.org/vert_71/). Then, the luciferase reporters were obtained via inserting the WT or MUT circ_0008305 and BAG5 3′‐UTR sequences into the pMIR‐REPORT Luciferase vector (Promega). Cells were co‐transfected with miR‐660 mimics, inhibitors, or control and the corresponding reporter plasmid using Lipofectamine 3000 (Invitrogen). After 24 h, the luciferase activity was determined using the Dual‐Luciferase reporter 1000 Assay System.

### 2.10 Tumor xenograft

Twelve female BALB/c mice (4‐week‐old females) were obtained from SILAC. 5 × 10^6^ HepG2 cells with or without transfection of circ_0008305 siRNA in 100 µL PBS were subcutaneously injected into the flanks of 6‐week‐old mice. After cell implantation, tumor size was recorded and measured every week from day 7 to day 42. The animals were sacrificed on day 42. All the experiments were performed following the NIH guidelines on animal welfare.

### 2.11 Immunohistochemistry staining

Xenograft tumors were cut into paraffin‐embedded sections. Afterwards, the slides were indicated with drying, deparaffining, and rehydrating. Slides were immersed with 3% hydrogen peroxide and labeled with Ki‐67 antibodies (Cell Signaling Technology) for a whole night. Next day, the slides were stained using the secondary antibody conjugated by horseradish peroxidase (Cell Signaling Technology).

### 2.12 Statistical analysis

We analyzed the data using SPSS 19.0 software (IBM) or GraphPad Prism 6 (GraphPad) and indicated as mean ± SD. Student's *t*‐test or one‐way ANOVA were employed to assess the statistic differences. *p* value less than 0.05 indicated a statistical significance.

## 3 RESULTS

### 3.1 Upregulation of circ_0008305 in HCC

**FIGURE 1:**
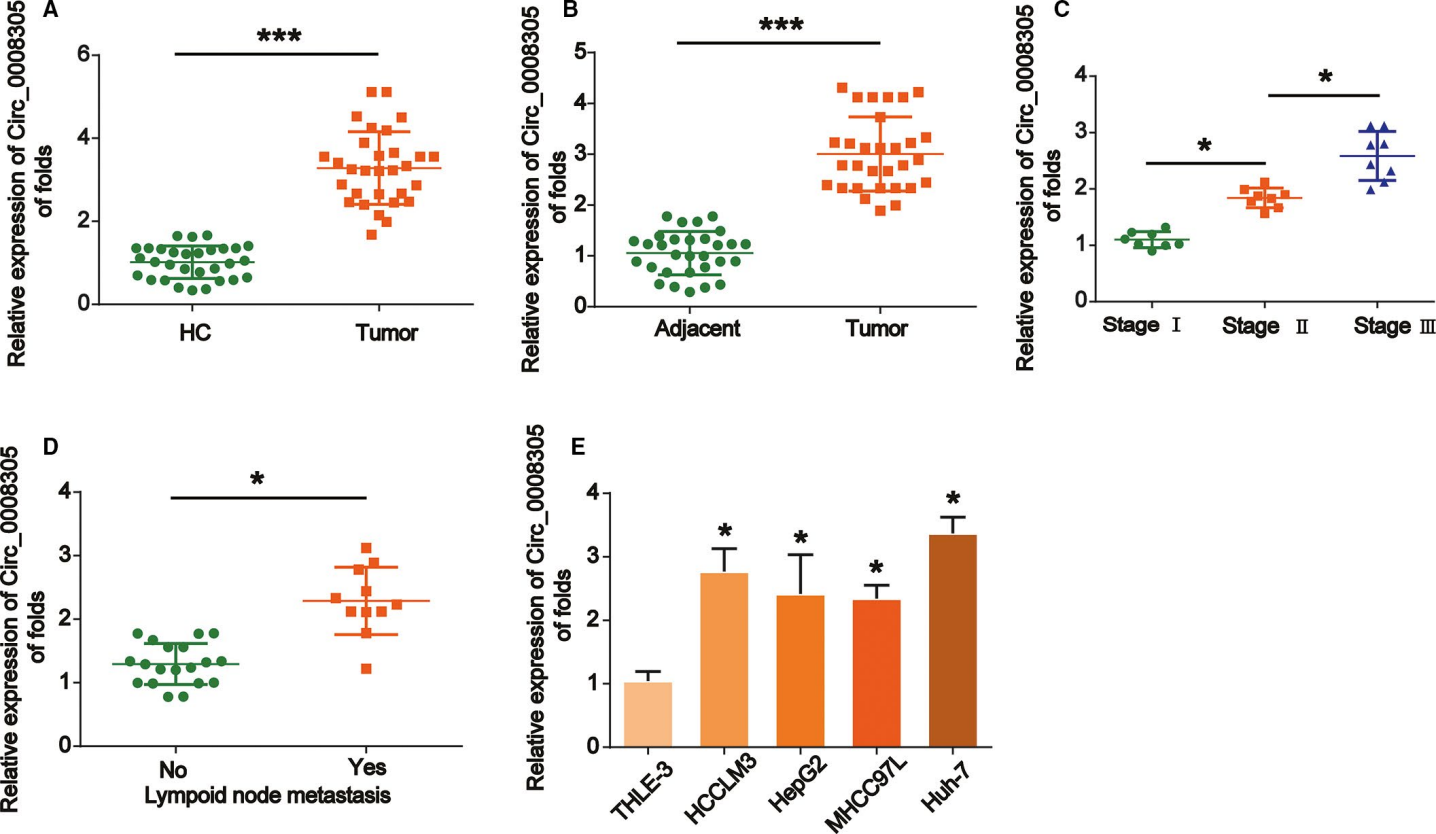
Circ_0008305 upregulation in HCC specimens and HCC cells. (A) Circ_0008305 expression was increased in HCC tissues compared to HC tissues. qRT‐PCR was performed to test the expression of circ_0008305 in normal tissues and tumor tissue. (B) Circ_0008305 expression was elevated in HCC tissues compared to adjacent tissues as evaluated using qRT‐PCR. (C) Circ_0008305 expression was higher in advanced HCC tissues. (D) Circ_0008305 expression was increased in metastatic HCC tissues. (E) Circ_0008305 levels were upregulated in HCC cell lines. Three independent experiments were carried out. Error bars stand for the mean ± SD of at least triplicate experiments. ***p < 0.001, *p < 0.005

To explore the function of circ_0008305 in HCC, its expression was determined in our work. Through using qRT‐PCR assay, we detected the expression level of circ_0008305 in normal liver tissues (HC, *n* = 30) and HCC tissues (*n* = 30). As shown in Figure 1A, we detected the expression level of circ_0008305 in normal liver tissues (HC, *n* = 30) and HCC tissues (*n* = 30). In Figure 1A, circ_0008305 was obviously increased in HCC tissues compared to the healthy controls. In addition, it was identified that circ_0008305 expression was upregulated in HCC tissues in comparison to the paired adjacent tissues (Figure 1B , *n* = 30). Then, we concentrated on the correlation between circ_0008305 and HCC progression. It was indicated that circ_0008305 expression was elevated in stage II–III of HCC tissues compared to stage I (Figure 1C). In addition, it was displayed in metastatic samples that upregulated levels of circ_0008305 were observed (Figure 1D). Moreover, consistently, we observed that circ_0008305 was also increased in HCC cancer cells (HCCLM3, HepG2, MHCC‐97L, and Huh‐7 cells) compared to THLE‐3 cells (Figure 1E). These indicated that circ_0008305 was upregulated in HCC.

### 3.2 Silencing circ_0008305 regulated HCC cell growth, apoptosis, and cell cycle.

**FIGURE 2:**
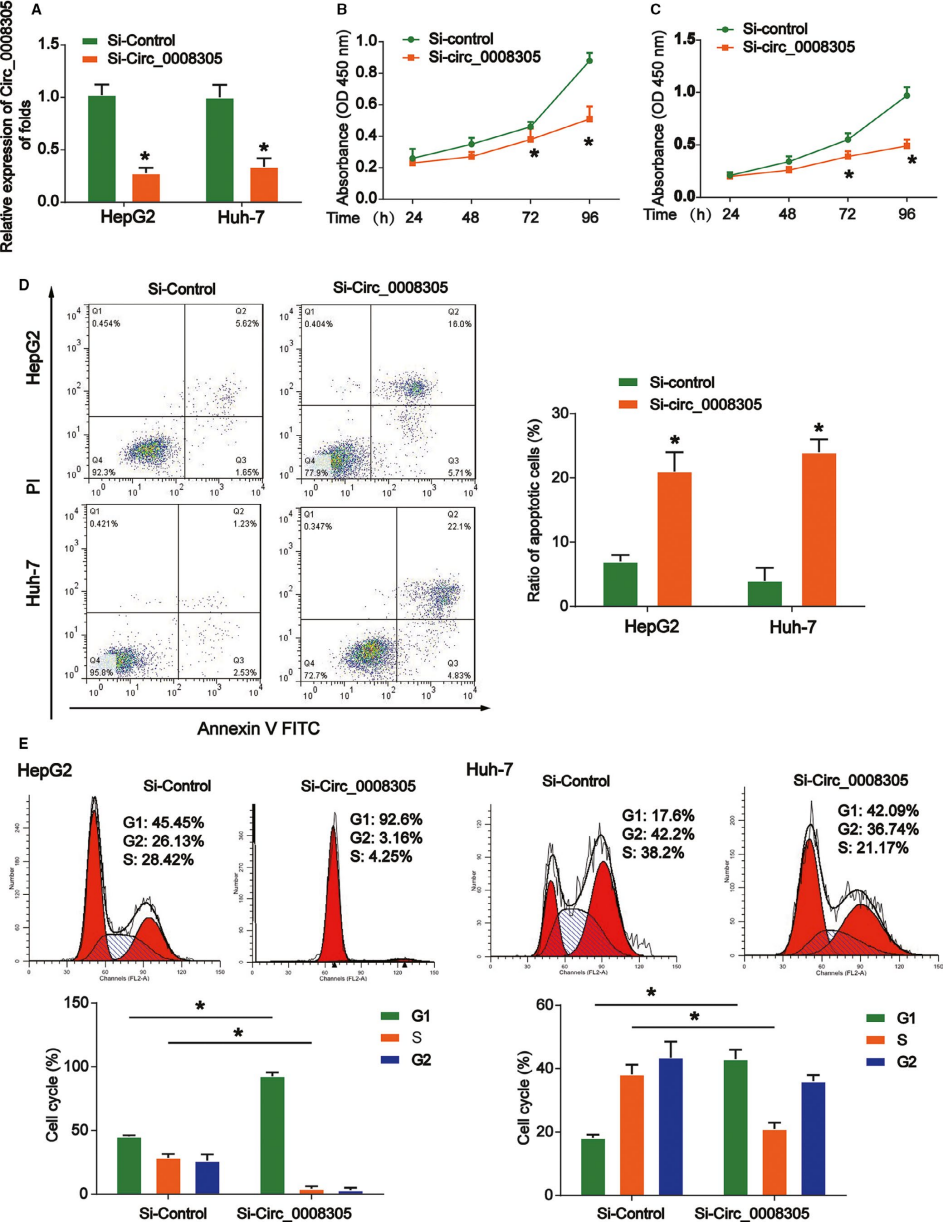
Silencing circ_0008305 regulated HCC cell proliferation, cell apoptosis and cell cycle. (A) qRT‐PCR analysis indicated that circ_0008305 knockdown led to decreased expression of circ_0008305 in HepG2 and Huh‐7 cells. (B and C) CCK8 assay illustrated that circ_0008305 silence reduced cellular proliferation. (D) Circ_0008305 knockdown promoted cellular apoptosis in HepG2 and Huh‐7 cells. Flow cytometry assay was used to test cell apoptosis. (E) Cell cycle distribution was measured using flow cytometry assay in HepG2 and Huh‐7 cells transfected with circ_0008305 siRNA. Three independent experiments were carried out. Error bars stand for the mean ± SD of at least triplicate experiments. *p < 0.05

Then, we studied the functions of circ_0008305 in HCC cells. Circ_0008305 was silenced in HepG2 and HUH‐7 cells. We proved the transfection efficiency and we found circ_0008305 was greatly decreased by circ_0008305 siRNA (Figure 2A). Next, CCK‐8 assay was conducted and it was displayed that circ_0008305 knockdown significantly suppressed HepG2 and HUH‐7 cell viability as exhibited in Figure 2B and C. Next, flow cytometry was utilized to analyze the influence of circ_0008305 on HCC cell apoptosis and cell cycle. Circ_0008305 silence triggered the apoptosis of HCC cells (Figure 2D). Additionally, HCC cell cycle was arrested in G1 phase and the cell ratio in S phase was reduced as exhibited in Figure 2E. These indicated that loss of circ_0008305 inhibited HCC cell growth.

### 3.3 Loss of circ_0008305 regulated HCC cell migration and invasion

**FIGURE 3:**
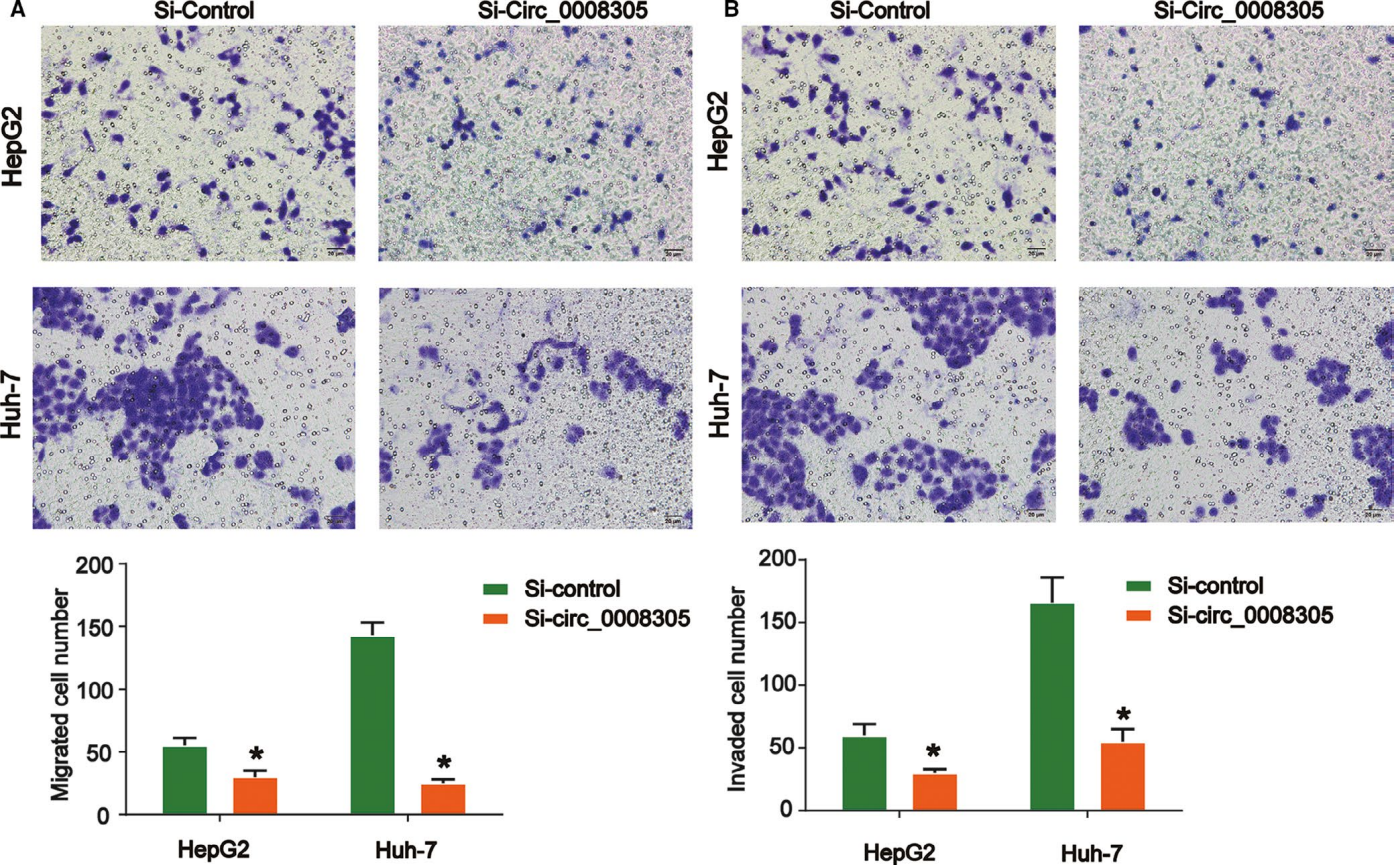
Loss of circ_0008305 regulated HCC cell migration and invasion capacity. (A) Effects of circ_0008305 siRNA on HepG2 and Huh‐7 cell migration. (B) Effects of circ_0008305 siRNA on HepG2 and Huh‐7 cell invasion. Cell migration and invasion abilities were evaluated by the transwell assay. Three independent experiments were carried out. Error bars stand for the mean ± SD of at least triplicate experiments. *p < 0.05

An association between circ_0008305 expression and metastasis in HCC was observed. Therefore, we carried out transwell assay to find out whether circ_0008305 regulated HCC metastasis. The data manifested that knockdown of circ_0008305 led to a reduced cell migration and invasion as demonstrated in Figure 3A and B. These indicated that silence of circ_0008305 restrained the migration and invasion abilities of HCC cells in vitro.

### 3.4 Decreased circ_0008305 reduced HCC tumor growth in vivo

**FIGURE 4:**
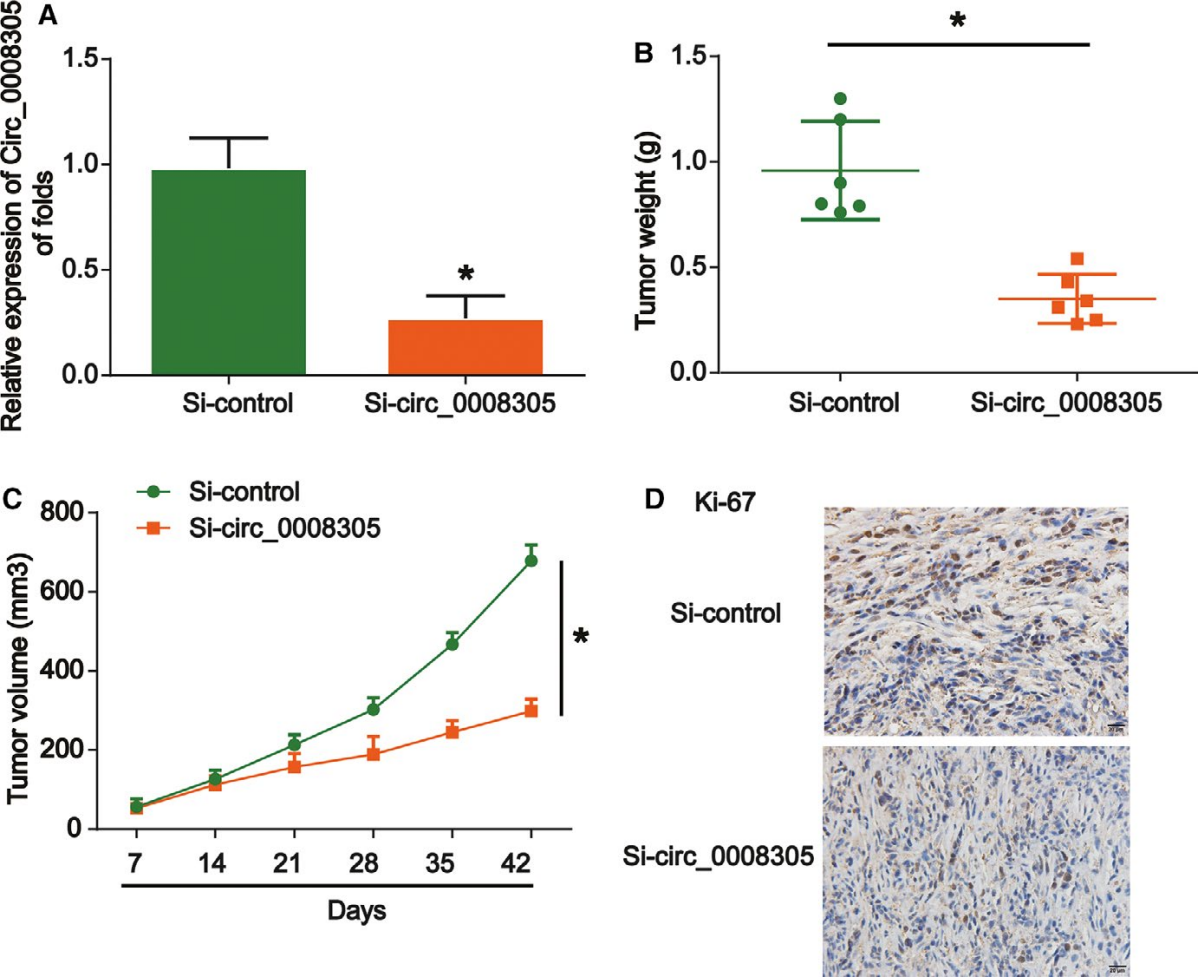
Knockdown of circ_0008305 inhibited tumor growth in vivo. Twelve 8‐week‐old female BALB/c nude mice were injected with HepG2 cells infected with shRNA‐control (six mice) or circ_0008305 siRNA (six mice). (A) The expression of circ_0008305 in the resected tumors was evaluated by qRT‐PCR. (B) Tumor weight. (C) Tumor volume in a time‐dependent manner. (D) IHC staining of Ki‐67 in tumor tissues. Three independent experiments were carried out. Error bars stand for the mean ± SD of at least triplicate experiments. *p < 0.05

Next, in order to better prove the role of circ_0008305, we carried out xenograft assays. Circ_0008305‐depleted or control HepG2 cells were injected into the mice and we recorded the tumor volumes every 7 days. Circ_0008305 was obviously decreased in circ_0008305‐depleted group in Figure 4A. Tumor weight was repressed by loss of circ_0008305 as exhibited in Figure 4B. In addition, we observed that knockdown of circ_0008305 markedly delayed HepG2 cell propagation (Figure 4C). In Figure 4D, IHC data of Ki‐67 indicated that circ_0008305 downregulation repressed HCC growth. These indicated that downregulation of circ_0008305 inhibited HCC tumor growth in vivo.

### 3.5 Circ_0008305 promoted BAG5 expression via sponging miR‐660

**FIGURE 5:**
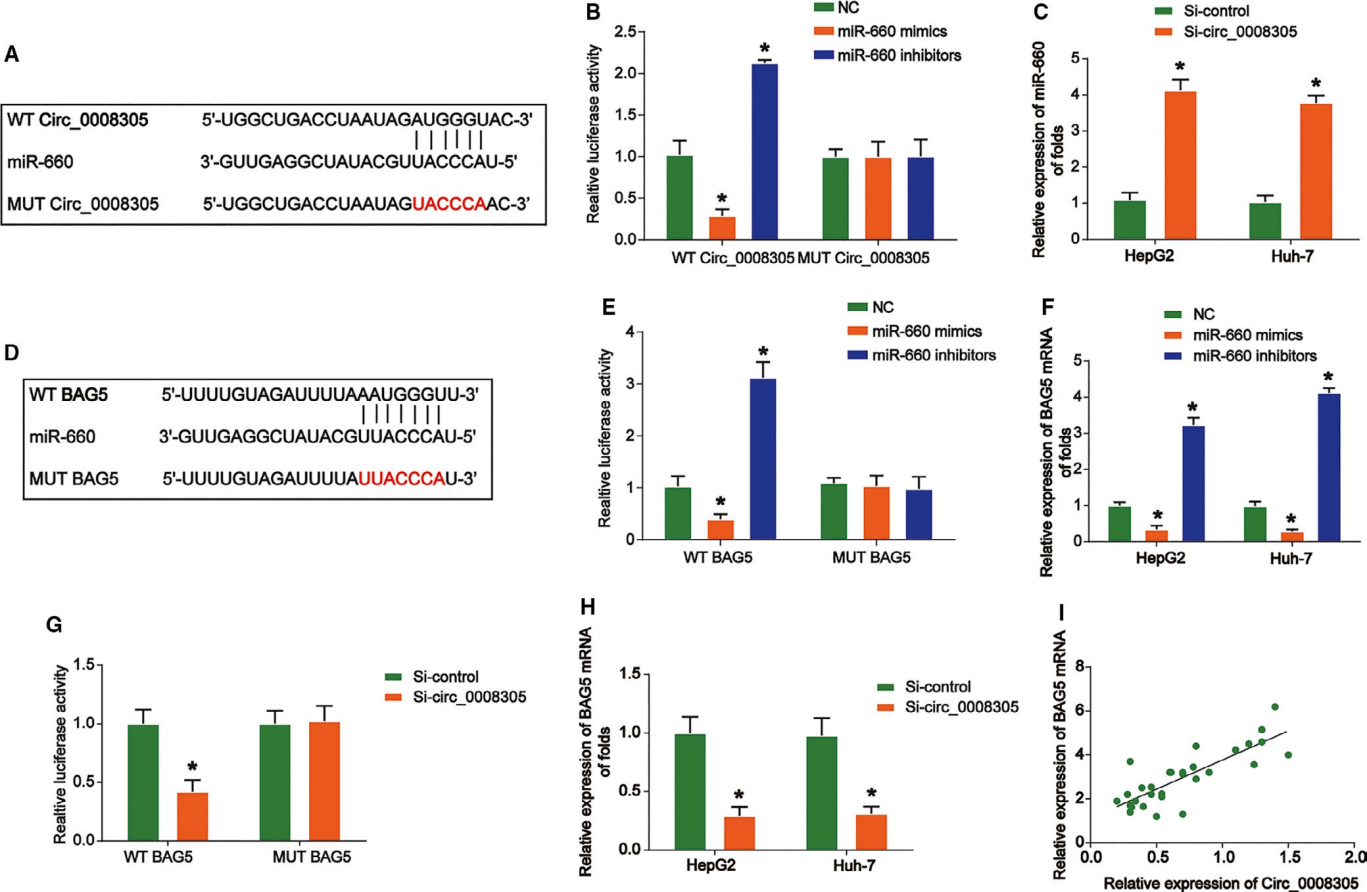
Circ_0008305 promoted BAG5 expression through sponging miR‐606. (A) Putative binding site for miR‐660 in circ_0008305 by bioinformatics identification. (B) Luciferase reporter assay showed miR‐660 mimics and inhibitors suppressed the activity of circ_0008305‐wild‐type (WT). (C) Circ_0008305 knockdown led to increased expression of miR‐660 in HepG2 and Huh‐7 cells. (D) Putative binding site for miR‐660 in BAG5 3’UTR. (E) Luciferase reporter assay showed miR‐660 mimics and inhibitors regulated the activity of BAG5‐WT. (F) miR‐660 mimics and inhibitors regulated BAG5 expression in HepG2 and Huh‐7 cells. (G) Luciferase reporter assay showed that circ_0008305 knockdown led to impaired luciferase activity of BAG5‐WT. (H) Circ_0008305 knockdown led to decreased expression of BAG5 in HepG2 and Huh‐7 cells. (I) Positive expression correlation between BAG5 and circ_0008305 or LARP1 in HCC tissues were determined in tumor tissue by Pearson's correlation analysis. Three independent experiments were carried out. Error bars stand for the mean ± SD of at least triplicate experiments. *p < 0.05

An increasing number of evidences reports circRNAs could act as miRNA sponge to modulate genes.^7^ To investigate the functional mechanism of circ_0008305, bioinformatics prediction was utilized on https://circinteractome.nia.nih.gov/. It was shown that miR‐660 might be targeted by circ_0008305. The putative binding sites in circ_0008305 for miR‐660 were shown in Figure 5A. Luciferase reporter assay proved miR‐660 mimics transfection repressed circ_0008305‐WT reporter luciferase activity in HepG2 cells while miR‐660 inhibitors exhibited a reversed process (Figure 5B), which could support a direct interaction between circ_0008305 and miR‐660. For another, circ_0008305 loss increased the expression of miR‐660 in HCC cells (Figure 5C). In addition, microRNAs are proven to be able to target mRNA 3’‐UTR.^15^ Therefore, we predicted BAG5 as the target gene of miR‐660 using http://www.targetscan.org/vert_71/. 3’‐UTR regions of BAG5 contained a potential binding region for miR‐660 (Figure 5D). Meanwhile, luciferase reporter assay indicated that miR‐660 reduced the luciferase activity of BAG5‐WT reporter in Figure 5E, which can indicate that miR‐660 interacted with BAG5. Additionally, miR‐660 modulated expression of BAG5 in HCC cells in Figure 5F. Then, we sought to find out whether circ_0008305 regulated the BAG5 expression. Furthermore, luciferase reporter assay evidenced circ_0008305 silence decreased the luciferase activity of BAG5‐WT reporter (Figure 5G), which suggested circ_0008305 regulates BAG5 expression via inhibiting miR‐660. Next, it was demonstrated that circ_0008305 silence depressed the BAG5 expression in HCC cells in Figure 5H. Notably, a positive expression correlation between BAG5 and circ_0008305 in HCC tissues was displayed (Figure 5I), convincingly proved that circ_0008305 inhibited miR‐660 while miR‐660 targeted BAG5. These indicated that circ_0008305 regulated the BAG5 expression through sponging miR‐660.

### 3.6 Circ_0008305 regulated HCC progression through modulating miR‐660/BAG5 pathway

**FIGURE 6:**
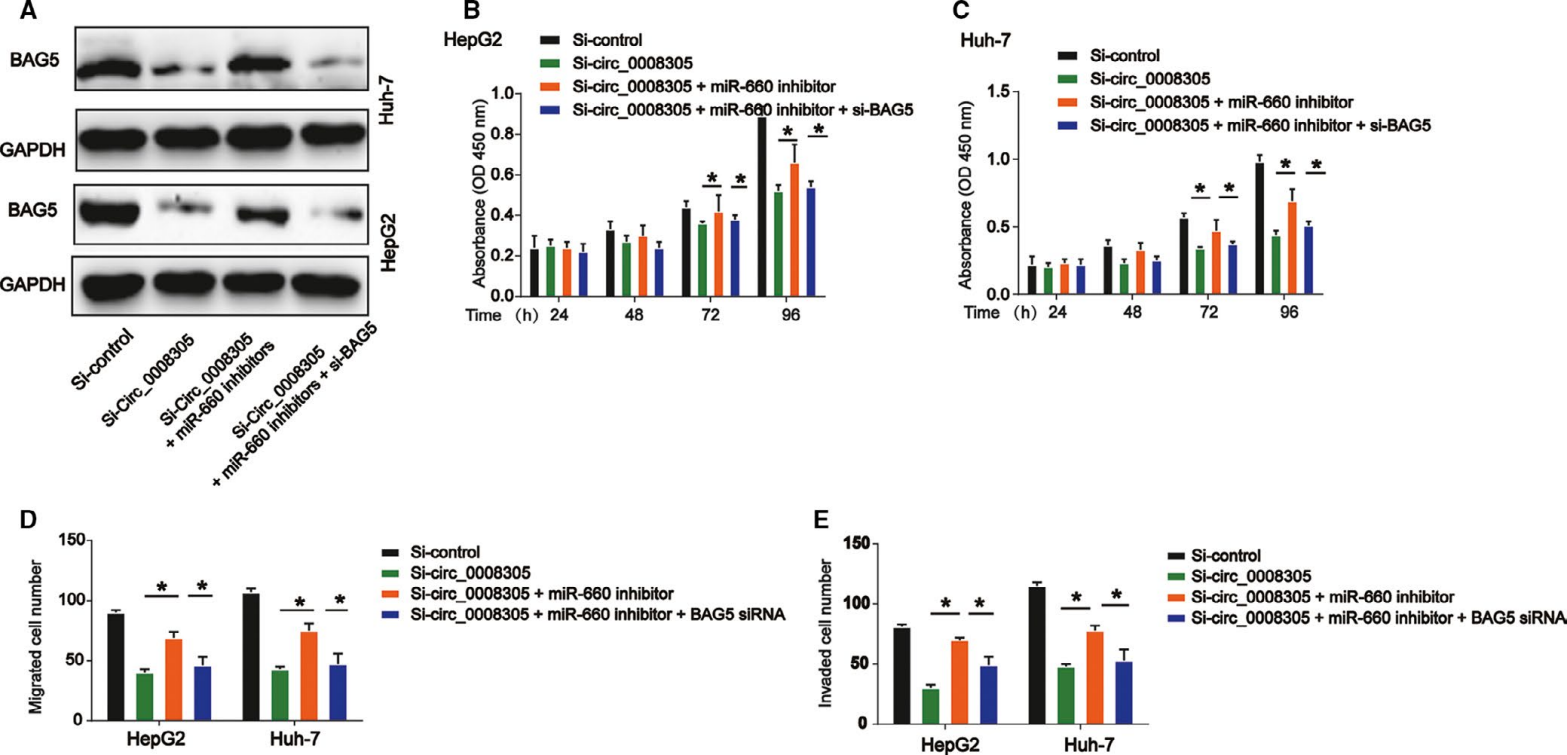
Circ_0008305 regulated HCC cell proliferation, migration and invasion by modulating miR‐660/BAG5 pathway. HepG2 and Huh‐7 cells were transfected with si‐control, circ_0008305 siRNA, miR‐144‐3p inhibitor, or BAG5 siRNA. (A) Relative protein levels of BAG5 in HepG2 and Huh‐7 cells. (B and C) CCK‐8 assay was used to determine cell proliferation in HepG2 and Huh‐7 cells. (D and E) Transwell assay was utilized to examine cell migration and invasion in HepG2 and Huh‐7 cells. Three independent experiments were carried out. Error bars stand for the mean ± SD of at least triplicate experiments. *p < 0.05

To figure out whether circ_0008305 functions through regulating miR‐660/BAG5 pathway, we carried out rescue assays via transfection with miR‐660 inhibitors or BAG5 siRNA in HepG2 and Huh‐7 cells. The transfection efficiency was validated via analyzing BAG5 protein levels in Figure 6A, followed by CCK8 and transwell assays. As demonstrated, loss of circ_0008305 decreased HCC cells proliferation, migration and invasion capacity (Figure 6B–E). However, miR‐660 inhibition increased HCC cell growth, whereas BAG5 knockdown reduced that (Figure 6B–E). These demonstrated circ_0008305 and BAG5 could exert tumor oncogenic roles while miR‐660 acted as a tumor suppressor in HCC. These indicated that circ_0008305 exerted its roles through modulating miR‐660/BAG5 axis.

## 4 DISCUSSION

CircRNAs are significant modulators in many pathological processes.^16^ Increasing evidence has indicated circRNA expression is closely co‐correlated with cancer progression.^17^, ^18^, ^19^ Thus, it is significant to evaluate the relationship between circRNAs and tumor progression. For instance, circRHOT1 can promote HCC progression via initiating NR2F6 expression.^20^ Circ_104075 can stimulate YAP‐dependent carcinogenesis via regulating HNF4a in HCC.^21^

Circ_0008305 has been reported to repress TGF‐β‐triggered EMT process through controlling TIF1γ in lung cancer.^22^ We identified that circ_0008305 can demonstrate its tumor oncogenic role in HCC. Our results showed circ_0008305 expression was obviously upregulated in HCC. It was also implied that loss of circ_0008305 suppressed HCC cell proliferation, induced cell apoptosis, blocked cell‐cycle progression, and repressed cell migration and invasion in vitro. In addition, it was showed that silencing circ_0008305 depressed HCC growth in vivo. Other circRNAs might also exert more important roles in HCC progression.

CircRNAs commonly act as specific sponges of microRNAs.^23^ To study the functional mechanism of circ_0008305, the potential target microRNAs were searched by us. miR‐660 was identified. Through carry out luciferase reporter assay, the association between circ_0008305 and miR‐660 in HCC cells was proved. Previously, it has been indicated that exosomal miR‐660‐5p can induce tumor progression in lung cancer.^24^ miR‐660 is sponged by circFBXL5 in promoting breast cancer progression.^25^ Here, we studied the role of miR‐660 in HCC. miR‐660 inhibitors reversed the effect of circ_0008305 in HCC progression. Whether other microRNAs are able to be related to circ_0008305 needs more investigation.

Accumulating studies have demonstrated that microRNAs exert roles via binding to the 3’‐UTR of their target mRNAs.^26^, ^27^ Here, through bioinformatics analysis, we showed miR‐660 might target BAG5. Via carrying out a series of assays, we validated their direct interaction. BAG5 belongs to BCL2 family and it is involved in translocations of BCL6 gene.^28^, ^29^ miR‐127‐3p functions as a tumor inhibitor in epithelial ovarian cancer through modulating BAG5.^30^ In addition, BAG5 is elevated in prostate cancer and represses ER‐stress triggered apoptosis.^31^ In addition, it has been shown that BAG5 can promote papillary thyroid cancer cell progression by upregulating fibronectin 1.^32^ However, how BAG5 expression was modulated in HCC is elusive. Our study pointed out that BAG5 was negatively correlated with circ_0008305 in HCC. Moreover, our data implied that BAG5 expression was modulated by circ_0008305/miR‐660 in HCC.

In summary, we first suggested that circ_0008305 was induced in HCC. Circ_0008305 was capable of serving as ceRNA for miR‐660 to regulate BAG5 level. Moreover, we displayed circ_0008305 demonstrated a significant role in HCC via regulating miR‐660/BAG5 axis.

## 5 ETHICS APPROVAL STATEMENT

This study was approved by the Medical Ethics Committee of Affiliated Hospital of Youjiang Medical University for Nationalities. All the animal experiments were approved by the Animal Ethics Committee of Affiliated Hospital of Youjiang Medical University for Nationalities, and our work was performed under the Guide for the Care and Use of Laboratory Animals of the National Institutes of Health.

## CONFLICT OF INTEREST

The authors declared no conflict of interest.

## Data Availability

All data are available upon request.

## References

[1] Torre LA , Bray F , Siegel RL , Ferlay J , Lortet‐Tieulent J , Jemal A . Global cancer statistics, 2012. CA Cancer J Clin. 2015;65:87‐108. 25651787 10.3322/caac.21262

[2] Jemal A , Bray F , Center MM , Ferlay J , Ward E , Forman D . Global cancer statistics. CA Cancer J Clin. 2011;61:69‐90. 21296855 10.3322/caac.20107

[3] El‐Serag HB . Hepatocellular carcinoma. N Engl J Med. 2011;365:1118‐1127. 21992124 10.1056/NEJMra1001683

[4] Sana J , Faltejskova P , Svoboda M , Slaby O . Novel classes of non‐coding RNAs and cancer. J Transl Med. 2012;10:103. 22613733 10.1186/1479-5876-10-103 PMC3434024

[5] Chen LL . The biogenesis and emerging roles of circular RNAs. Nat Rev Mol Cell Biol. 2016;17:205‐211. 26908011 10.1038/nrm.2015.32

[6] Yao R , Zou H , Liao W . Prospect of circular RNA in hepatocellular carcinoma: a novel potential biomarker and therapeutic target. Front Oncol. 2018;8:332. 30191143 10.3389/fonc.2018.00332 PMC6115511

[7] Verduci L , Strano S , Yarden Y , Blandino G . The circRNA‐microRNA code: emerging implications for cancer diagnosis and treatment. Mol Oncol. 2019;13:669‐680. 30719845 10.1002/1878-0261.12468 PMC6441890

[8] Meng S , Zhou H , Feng Z , et al. CircRNA: functions and properties of a novel potential biomarker for cancer. Mol Cancer. 2017;16:94. 28535767 10.1186/s12943-017-0663-2 PMC5440908

[9] Hansen TB , Jensen TI , Clausen BH , et al. Natural RNA circles function as efficient microRNA sponges. Nature. 2013;495:384‐388. 23446346 10.1038/nature11993

[10] Holdt LM , Stahringer A , Sass K , et al. Circular non‐coding RNA ANRIL modulates ribosomal RNA maturation and atherosclerosis in humans. Nat Commun. 2016;7:12429. 27539542 10.1038/ncomms12429 PMC4992165

[11] Zheng Q , Bao C , Guo W , et al. Circular RNA profiling reveals an abundant circHIPK3 that regulates cell growth by sponging multiple miRNAs. Nat Commun. 2016;7:11215. 27050392 10.1038/ncomms11215 PMC4823868

[12] Chen D , Ma W , Ke Z , Xie F . CircRNA hsa_circ_100395 regulates miR‐1228/TCF21 pathway to inhibit lung cancer progression. Cell Cycle. 2018;17:2080‐2090. 30176158 10.1080/15384101.2018.1515553 PMC6224268

[13] Yang CY , Zhang FX , He JN , Wang SQ . CircRNA_100876 promote proliferation and metastasis of breast cancer cells through adsorbing microRNA‐361‐3p in a sponge form. Eur Rev Med Pharmacol Sci. 2019;23:6962‐6970. 31486496 10.26355/eurrev_201908_18736

[14] Xie F , Li Y , Wang M , et al. Circular RNA BCRC‐3 suppresses bladder cancer proliferation through miR‐182‐5p/p27 axis. Mol Cancer. 2018;17:144. 30285878 10.1186/s12943-018-0892-z PMC6169039

[15] Mohr AM , Mott JL . Overview of microRNA biology. Semin Liver Dis. 2015;35:3‐11. 25632930 10.1055/s-0034-1397344 PMC4797991

[16] Hsiao KY , Sun HS , Tsai SJ . Circular RNA–new member of noncoding RNA with novel functions. Exp Biol Med. 2017;242:1136‐1141. 10.1177/1535370217708978 PMC5478007 28485684

[17] Patop IL , Kadener S . circRNAs in Cancer. Curr Opin Genet Dev. 2018;48:121‐127. 29245064 10.1016/j.gde.2017.11.007 PMC5877416

[18] Shang Q , Yang Z , Jia R , Ge S . The novel roles of circRNAs in human cancer. Mol Cancer. 2019;18:6. 30626395 10.1186/s12943-018-0934-6 PMC6325800

[19] Ng WL , Mohd Mohidin TB , Shukla K . Functional role of circular RNAs in cancer development and progression. RNA Biol. 2018;15:995‐1005. 29954251 10.1080/15476286.2018.1486659 PMC6259826

[20] Wang L , Long H , Zheng Q , Bo X , Xiao X , Li B . Circular RNA circRHOT1 promotes hepatocellular carcinoma progression by initiation of NR2F6 expression. Mol Cancer. 2019;18:119. 31324186 10.1186/s12943-019-1046-7 PMC6639939

[21] Zhang X , Xu Y , Qian Z , et al. circRNA_104075 stimulates YAP‐dependent tumorigenesis through the regulation of HNF4a and may serve as a diagnostic marker in hepatocellular carcinoma. Cell Death Dis. 2018;9:1091. 30361504 10.1038/s41419-018-1132-6 PMC6202383

[22] Wang L , Tong X , Zhou Z , et al. Circular RNA hsa_circ_0008305 (circPTK2) inhibits TGF‐beta‐induced epithelial‐mesenchymal transition and metastasis by controlling TIF1gamma in non‐small cell lung cancer. Mol Cancer. 2018;17:140. 30261900 10.1186/s12943-018-0889-7 PMC6161470

[23] Rong D , Sun H , Li Z , et al. An emerging function of circRNA‐miRNAs‐mRNA axis in human diseases. Oncotarget. 2017;8:73271‐73281. 29069868 10.18632/oncotarget.19154 PMC5641211

[24] Qi Y , Zha W , Zhang W . Exosomal miR‐660‐5p promotes tumor growth and metastasis in non‐small cell lung cancer. J BUON. 2019;24:599‐607. 31128012

[25] Zhou H , Tang G , Zhao MI , et al. circFBXL5 promotes breast cancer progression by sponging miR‐660. J Cell Mol Med. 2020;24:356‐361. 31729134 10.1111/jcmm.14737 PMC6933392

[26] O'Brien J , Hayder H , Zayed Y , Peng C . Overview of microRNA biogenesis, mechanisms of actions, and circulation. Front Endocrinol. 2018;9:402. 10.3389/fendo.2018.00402 PMC6085463 30123182

[27] Cannell IG , Kong YW , Bushell M . How do microRNAs regulate gene expression? Biochem Soc Trans. 2008;36:1224‐1231. 19021530 10.1042/BST0361224

[28] Horn H , Ziepert M , Becher C , et al. MYC status in concert with BCL2 and BCL6 expression predicts outcome in diffuse large B‐cell lymphoma. Blood. 2013;121:2253‐2263. 23335369 10.1182/blood-2012-06-435842

[29] Kramer M , Hermans J , Wijburg E , et al. Clinical relevance of BCL2, BCL6, and MYC rearrangements in diffuse large B‐cell lymphoma. Blood. 1998;92:3152‐3162. 9787151

[30] Bi L , Yang Q , Yuan J , et al. MicroRNA‐127‐3p acts as a tumor suppressor in epithelial ovarian cancer by regulating the BAG5 gene. Oncol Rep. 2016;36:2563‐2570. 27571744 10.3892/or.2016.5055

[31] Bruchmann A , Roller C , Walther TV , et al. Bcl‐2 associated athanogene 5 (Bag5) is overexpressed in prostate cancer and inhibits ER‐stress induced apoptosis. BMC Cancer. 2013;13:96. 23448667 10.1186/1471-2407-13-96 PMC3598994

[32] Zhang DL , Wang JM , Wu T , et al. BAG5 promotes invasion of papillary thyroid cancer cells via upregulation of fibronectin 1 at the translational level. Biochim Biophys Acta Mol Cell Res. 2020;1867:118715. 32275930 10.1016/j.bbamcr.2020.118715

